# National cost study versus hospital cost accounting for organ recovery cost assessment in a French hospital group

**DOI:** 10.1186/s12962-018-0155-2

**Published:** 2018-10-11

**Authors:** Abdelbaste Hrifach, Christell Ganne, Sandrine Couray-Targe, Coralie Brault, Pascale Guerre, Hassan Serrier, Hugo Rabier, Gwen Grguric, Pierre Farge, Cyrille Colin

**Affiliations:** 1 Pôle de Santé Publique, Service d’Evaluation Economique en Santé, Hospices Civils de Lyon, 162, Avenue Lacassagne - Bâtiment A, 69424 Lyon Cedex 03, France; 2 HESPER EA 7425, Univ. Lyon, Université Claude Bernard Lyon 1, 69008 Lyon, France; 3 Pôle de Santé Publique, Département d’Information Médicale, Hospices Civils de Lyon, 69424 Lyon, France; 4 Hospices Civils de Lyon, Cellule Innovation, Délégation à la Recherche Clinique et à l’Innovation, 69237 Lyon, France; 5 Univ. Lyon, Université Claude Bernard Lyon 1, 69008 Lyon, France

**Keywords:** Cost data source, Hospital cost accounting, National cost study, Organ recovery

## Abstract

**Background:**

The choice of cost data sources is crucial, because it influences the results of cost studies, decisions of hospital managers and ultimately national directives of policy makers. The main objective of this study was to compare a hospital cost accounting system in a French hospital group and the national cost study (ENC) considering the cost of organ recovery procedures. The secondary objective was to compare these approaches to the weighting method used in the ENC to assess organ recovery costs.

**Methods:**

The resources consumed during the hospital stay and organ recovery procedure were identified and quantified retrospectively from hospital discharge abstracts and the national discharge abstract database. Identified items were valued using hospital cost accounting, followed by 2010–2011 ENC data, and then weighted using 2010–2011 ENC data. A Kruskal–Wallis test was used to determine whether at least two of the cost databases provided different results. Then, a Mann–Whitney test was used to compare the three cost databases.

**Results:**

The costs assessed using hospital cost accounting differed significantly from those obtained using the ENC data (Mann–Whitney; P-value < 0.001). In the ENC, the mean costs for hospital stays and organ recovery procedures were determined to be €4961 (SD €7295) and €862 (SD €887), respectively, versus €12,074 (SD €6956) and €4311 (SD €1738) for the hospital cost accounting assessment. The use of a weighted methodology reduced the differences observed between these two data sources.

**Conclusions:**

Readers, hospital managers and decision makers must know the strengths and weaknesses of each database to interpret the results in an informed context.

## Background

The choice of a cost data source is important because, as demonstrated in the case of colorectal cancer, using different data sources can produce widely different estimates of health care costs [1, 2]. Currently, in a context of budget restrictions and trends toward more efficiency in resource allocation, choosing the most appropriate cost database for medico-economic evaluations appears to be a crucial determinant insofar as this choice influences not only the results of cost studies but also the decisions of hospital managers and ultimately the national directives of health policy makers [3]. In the absence of approved guidelines and recommendations, researchers and health economists conducting cost–benefit analyses, hospital internal investigations or national reports must choose between several cost data sources with the risk of consciously or unconsciously biasing the results [3].

Given the increasing need for and popularity of cost studies worldwide, the development of multiple cost databases to assess health care costs has raised difficulty in the choice of cost data sources. In the United States, Lund et al. [4] identified more than 80 data sources that could be used to estimate health care costs, including data aggregated at the patient, hospital and national levels. A major difficulty in applying some of the available databases to cost analyses (e.g., insurance databases) is that these databases were initially created and designed for reimbursement processes of hospital expenses and not for the measurement of health care costs [5]. Moreover, each data source represents a unique population and has its own level of aggregation, periodicity and access cost, which must be considered when selecting the most appropriate data source for each specific research question [4].

Since the introduction of diagnosis-related groups (DRGs) and the deployment of activity-based pricing, European countries have developed their own hospital cost accounting systems as a basis for better resource management and valuation of medical services [6]. The operating principle is based on cost accounting data of a sample of hospitals at a national and more aggregate level to calculate hospital costs, attribute a specific cost to each DRG and finally make a pricing decision for the payer [7]. In this model, the initial cost accounting of the hospital has an important place, because it provides the first cost data for valuation of medical services and sets their prices at the national level. France’s DRG system has some idiosyncrasies. The classification of each hospital stay in a medical or surgical DRG is related to the main diagnosis for admission and the presence or not of a surgical classification procedure in the discharge abstracts. Organ recovery is not a classified procedure; thus, the hospital stay can be distributed among surgical or medical DRGs. DRGs are a medico-economic classification system that pools together hospital stays with medical and economic homogeneity. The activity-based pricing is a hospital funding method based on resource allocation according to the volume and nature of their activities.

In France, the health ministry has used financial incentives to encourage implementation of a standardized cost accounting system in hospitals [8, 9]. Hospital costs per stay are calculated using a top-down costing method that combines medical and nonmedical cost data for each stay. Despite a few particularities, the sample of French hospitals that participated in the national cost study (ENC) used the same model of cost accounting and transferred their cost data from the hospital database to the national level in the ENC [10].

The few studies that have analyzed the impact of different data sources on cost care assessment often compare a study group to a control group matched by sex, age, geographic location and other parameters [1, 2]. However, no study has compared the hospital costs of the same patients using different data sources. For example, in 2016, the kidney recovery cost was assessed from a French hospital cost accounting system at €5439 [11]. In a more recent publication based on the ENC, the kidney recovery cost was assessed at €1432 [12]. Despite the increasing importance of organ recovery and the internationalization of this public health issue, few studies have focused on this problem. Given that organ recovery cost assessment has been sparsely investigated and that the first results issued from different data sources appear to differ widely, we decided to compare the costs of organ recovery procedures for the same patients from different cost data sources, with each source representing a different level of data aggregation.

The main objective of this study was to compare a hospital cost accounting system in a French hospital group with the ENC in terms of the cost of organ recovery procedures. The secondary objective was to compare both approaches to a previously described weighting method [12] to assess organ recovery costs when the ENC was used.

## Methods

### Study design

This study was based on discharge data from organ recovery performed in the public hospital group ‘*Hospices Civils de Lyon’* (HCL) from January 2010 to December 2011. Direct medical costs were estimated from the hospital’s perspective. Direct nonmedical costs and indirect costs were not considered in this study. The timeframe considered was from the beginning until the end of the hospital stay during which the organ recovery was performed. Costs related to family management, liquid preservation, machine perfusion and organ shipment were excluded, because they were not directly related to the surgical procedure.

### Study population

All brain death donors who underwent kidney, liver, pancreas, intestine, heart, lung or heart–lung block recovery in the HCL between January 2010 and December 2011 were eligible for the analysis. To compare costs for the same donors according to the hospital cost accounting and ENC data, we established a patient selection algorithm that combined the hospital identification code, year of hospitalization, patient age, patient sex, patient DRG and number of procedures performed. Donors were excluded if the selection algorithm could not match them in both databases with certainty. Living donors and donors after circulatory death were also excluded.

### Cost data sources

The HCL cost accounting system and the 2010 and 2011 ENCs were used for this analysis. To respond to the second objective, weighted ENCs were also used. As hospitals participating in the ENC, the HCL consider the same items as the ENC in the process of cost identification, which enables comparison of the valuation realized in hospital cost accounting at the local level with that realized in the ENC at the national level.

### HCL cost accounting system

For each deceased donor whose organs were recovered during the two consecutive years in the HCL, a discharge abstract combining personal and stay data was identified retrospectively in a local database. Cost data related to each hospital stay were collected by the HCL cost accounting system, which represented the first and the local source of economic information used for hospital management and economic valuation of medical services.

### Enc

Hospitals participating in the ENC send elements of their own cost accounting, their activity and the follow-up of expenses during the stay to the national level. Then, the central authority conducts a first treatment on the collected data that consists of eliminating hospital stays with atypical costs corresponding to an error in the allocation of the charges or the medical coding. The central authority applies a methodology for discharging expenses for stays and ultimately produces a full cost per stay (including staff costs, drugs, technical acts, logistics, fees and other costs). The cost data collected at the national level in the ENC from a panel of public and private health institutions allowed us to value each hospital stay corresponding to organ recovery during these two consecutive years in the HCL. In contrast to hospital cost accounting, which addresses the original hospital stay costs, the costs collected in the ENC are retrieved to obtain national average costs per homogeneous groups of patients that are published annually [13, 14].

### Weighted ENC

Because organ recovery is not a procedure that is classified in a specific DRG, hospital stays for organ recovery may correspond to either a medical DRG or a surgical DRG in the ENC according to the main reason for hospital admission. Because the medical DRG entails the risk of greatly underestimating the costs of surgical procedures, a weighting method previously described in the literature was applied to revalue the ENC data [12].

### Identification and quantification of cost components

The resources consumed during the hospital stay and the organ recovery procedure were identified and quantified retrospectively from the hospital discharge abstracts and national discharge abstract database. The eight items identified were surgery, anesthesia, reanimation, intensive care, ongoing monitoring, biology, imaging and medical logistics. Reanimation, intensive care and ongoing monitoring are grouped into the critical care item. All items except medical logistics were subdivided into 5 subitems (medical staff, nonmedical staff, nursing staff, maintenance, depreciation and cost of block occupation). Medical logistics items were regrouped into sterilization, biomedical engineering, hygiene, vigilance and pharmacy.

### Valuation of cost components

All cost components were valued in euros at 2011 prices. The items identified were first valued using hospital cost accounting, followed by 2010 and 2011 ENC data, and then weighted using the 2010 and 2011 ENC data. All three methods use a top-down microcosting approach as the costing method. Top-down microcosting identifies all relevant hospital services at the most detailed level but values each hospital service per average patient [15, 16]. Because top-down microcosting does not require patient-level data, statistical analyses of costs cannot be performed, and differences between patients cannot be detected [15]. However, in France, organ recovery is not a procedure that is classified in a specific DRG. Thus, for the same organ recovery procedure, donors are affiliated with different groups of patients in the local database and with different DRGs in the national database according to different parameters. The variability in donor distribution facilitates statistical analyses to determine whether cost valuation differences exist among HCL cost accounting, ENC and weighted ENC data for the same hospital stays.

In all three cost valuation methods, each surgical procedure related or unrelated to organ recovery is characterized by relative cost indexes (RCIs). A RCI is used to assess the cost of a procedure performed in ideal conditions [17]. These indexes are commonly used to break down the overall cost of surgical activities according to the number of RCIs specific to each procedure [18]. For each donor, we assessed a ratio corresponding to the portion of RCIs related to organ recovery out of the total number of RCIs related to surgical activities. Then, surgery, anesthesia, biology, imaging and medical logistics were valued from the cost data weighted by the ratio assessed as related exclusively to organ recovery. Regarding reanimation, intensive care and ongoing monitoring, discussions with hospital coordinators of organ and tissue procurement allowed us to elaborate the working hypothesis that expenditures related to organ recovery were exclusively focused on the last day of the hospital stay. Thus, reanimation, intensive care and ongoing monitoring were valued from the cost data weighted by the length of stay corresponding to each donor. The organ recovery procedure was assessed with the same approach to reveal the cost differences among the hospital cost accounting, ENC and weighted ENC data.

### Analyses

Three cost evaluations were conducted, each of which explored a specific characteristic of the organ recovery procedures. All costs are presented according to their means and standard deviations (SDs).

- The cost of hospital stays during which an organ recovery procedure was performed was obtained for an overview of hospital costs related to organ recovery activity.
- The cost of organ recovery procedures was obtained to assess the portion of the hospital stay costs related exclusively to organ recovery activities.
- The costs of the eight items identified were also specified to identify whether the cost of one or more items differed more widely among the data sources.

A Kruskal–Wallis test was used to detect whether at least two of the cost databases provided different results. Then, a Mann–Whitney test was used to compare “hospital cost accounting” versus ENC, “hospital cost accounting” versus weighted ENC and ENC versus weighted ENC.

## Results

From January 2010 to December 2011, 103 and 101 brain death donors were identified in the local and national databases, respectively. The selection algorithm matched 88 of these donors for inclusion in the analysis, corresponding to a total of 201 organs recovered (Table 1).

**Table 1:** Deceased donor characteristics and practices between January 2010 and December 2011 in the HCL

	Donors	Age	Number of organs recovered simultaneously						Organs/donor
			1	2	3	4	5	> 5	
2010	54	52	17	19	9	7	2	0	2.22
2011	34	48	10	10	9	1	4	0	2.38
Total	88	51	27	29	18	8	6	0	2.28

The Kruskal–Wallis test highlighted the differences among the three cost data sources in the cost of hospital stay, the cost of organ recovery and all items of the organ recovery procedure except for imaging (Table 2). Thus, a Mann–Whitney test was applied to compare the three cost databases in terms of all costs except imaging costs (Table 2).

**Table 2:** Organ recovery cost assessment using different cost data sources in the HCL between 2010 and 2011 (2011 euros)

	(1) Hospital cost accounting	(2) National cost study	(3) Weighted national cost study	Kruskal–Wallis	Mann–Whitney		
	Mean (SD)	Mean (SD)	Mean (SD)	P-value	∆1–2	∆1–3	∆2–3
Hospital stay	12,074 (6956)	4961 (7295)	4961 (7295)	< 0.001	< 0.001	< 0.001	1
Recovery procedure	4313 (1 738)	862 (887)	1490 (753)	< 0.001	< 0.001	< 0.001	< 0.001
Surgery	1080 (573)	112 (198)	468 (146)	< 0.001	< 0.001	< 0.001	< 0.001
Anesthesia	975 (494)	91 (142)	362 (111)	< 0.001	< 0.001	< 0.001	< 0.001
Critical care	1202 (705)	350 (447)	350 (447)	< 0.001	< 0.001	< 0.001	1
Imaging	188 (275)	108 (98)	108 (98)	0.671	NA	NA	NA
Biology	453 (616)	116 (117)	116 (117)	< 0.001	< 0.001	< 0.001	1
Logistics	415 (202)	86 (110)	86 (110)	< 0.001	< 0.001	< 0.001	1

### Comparison of hospital cost accounting valuation with ENC assessment

The costs assessed using hospital cost accounting differ significantly from those assessed using ENC data (Mann–Whitney; P-value < 0.001).

Using hospital cost accounting, the mean costs for the hospital stay, recovery procedure, surgery, anesthesia, critical care, biology and logistics were determined to be €12,074 (SD €6956), €4311 (SD €1738), €1080 (SD €573), €975 (SD €494), €1202 (SD €705), €453 (SD €616) and €415 (SD €202), respectively (Table 2).

Using the ENC as the data source, the costs assessed for the same donors and the same items were significantly lower, and the SDs increased considerably (Mann–Whitney; P-value < 0.001). The mean costs for the hospital stay, recovery procedure, surgery, anesthesia, critical care, biology and logistics were determined to be €4961 (SD €7295), €862 (SD €887), €112 (SD €198), €91 (SD €142), €350 (SD €447), €116 (SD €117) and €86 (SD €110), respectively (Table 2).

### Comparison of the weighted ENC assessment with the ENC and hospital cost accounting valuations

The data revaluation of the weighted ENC exclusively concerned the surgery and anesthesia items, as previously described [12]. This revaluation decreased the gap between the cost assessments based on hospital cost accounting and the original ENC data. Nevertheless, the cost assessment based on the weighted ENC remained significantly lower than the assessment based on hospital cost accounting data regardless of the cost evaluation performed (Mann–Whitney; P-value < 0.001) (Table 2).

No differences in critical care, biology and logistics were observed between the assessments based on the weighted ENC and the original ENC, because the revaluation method did not concern these items (Table 2).

## Discussion

In France, the standardization policies for hospital cost accounting and the ENC allow item-by-item comparisons of hospital costs related to organ recovery from different levels of data aggregation. Implementation of a selection algorithm combining patient parameters could facilitate comparison of the same patients and the same hospital stays. Using the ENC, the mean costs for hospital stays and organ recovery procedures were determined to be €4961 (SD €7295) and €862 (SD €887), respectively, versus €12,074 (SD €6956) and €4311 (SD €1738) for the hospital cost accounting assessment. The use of the ENC seemed to underestimate the cost valuation compared to valuation by hospital cost accounting. Use of the weighted ENC methodology to better reflect the organ recovery cost decreased the differences between hospital cost accounting and the ENC. Despite use of this weighting method, cost differences remained among the three data sources.

As the first economic evaluation conducted in France to compare several cost data sources, this study highlights existing cost differences between hospital cost accounting and the French ENC. These differences relativize and lend caution to interpretation of the results of our previous study, which assessed organ recovery costs from ENC data [12]. Generally, the current findings question the use of the ENC as a reference for economic evaluations. The multiple and complex reprocessing operations prevent the ENC from providing a real national mean cost for hospital services [19]. Currently, the ENC data may more usefully reflect a distribution of a global healthcare budget across different DRGs and may not reflect real hospital costs based on hospital cost accounting [19]. Nevertheless, the ENC data remain important and are widely used, and the economic information based on the ENC facilitates resource management for healthcare providers without their own cost accounting systems [20].

Most authors agree that no perfect cost database exists for all research questions. Many of the currently available data sources, including administrative sources, have not been designed for medico-economic assessments [21]. In the absence of guidelines, the authors recommend choosing the data source according to the study purpose and timing [21]. The strengths and weakness of the chosen data source should be kept in mind and clearly debated. However, few studies have tested the impact of the data source on cost assessment. The case of colorectal cancer strengthens our results. In fact, Yabroff et al. [1] showed that the mean net annual per person cost varied significantly from $5341 to $11,614 according to the data source chosen. These authors affirmed that no gold standard data source existed for estimation of the prevalence costs of cancer care. In 2009, Lund et al. [4] showed that different levels of data aggregation were present among the 88 data sources referenced in the US, which may influence the cost analysis results.

Our results are comparable to those of Fagnoni et al. [22], who found that the costs of managing acute myeloid leukemia were 2 to 4 times higher in the CAH than in the ENCC. Conversely, our results differ from those presented by Chaumard et al. [23], who found that the hospital stay cost for a renal transplant was significantly lower with the CAH estimate than with the ENC estimate. These different results reflect that depending on the pathology or care considered, some GHMs are less well valued in the ENC than in the CAH. For a given hospital, Chaumard et al. [23] hypothesized that favorable GHMs would globally offset adverse GHMs. This hypothesis can be valid in the case of hospitals with a varied activity panel and a wide variety of GHMs. In reality, the French healthcare system includes multidisciplinary hospitals and hospitals with a specialized activity characterized by a reduced number of GHMs. Moreover, a certain number of establishments are orienting their activities toward remunerative GHMs to the detriment of unfavorable GHMs with a view toward profitability.

Zeynep Or demonstrated that the type of establishment influenced the hospital stay cost [24]. The author noted that in most situations, additional costs were incurred in university hospital centers. As one such center, the HCL is particularly exposed to these additional costs, which reflects the specific characteristics of university hospital centers that include research and teaching activities. The absence of patient selection and the admission of more serious cases into these institutions also impact the cost of a university hospital stay [25, 26]. In fact, patients are not randomly distributed among hospitals, and some hospitals consistently receive more patients associated with higher costs for the same DRG [27]. Moreover, hospital costs were demonstrated to vary according to the size and volume of the activity [28–30]. As a university hospital with a large volume of activity, the HCL is particularly exposed to scale “diseconomies”. In 1996, Rosko demonstrated that beyond a certain threshold, increasing the volume of production rather than achieving economies of scale leads to increased costs due to coordination and organizational problems [31]. All of these elements can explain the differences observed between valuation based on HCL’s cost accounting and that based on ENC data.

Some limitations of our work should be noted. Although the number of hospital stays was limited to cases of a single public institution, the HCL was chosen based on its several years of regular participation in the ENC, which reflected its engagement in data collection. Extension of this comparative study to other hospitals and the inclusion of a longer observation period would strengthen our results concerning the status of the ENC as a cost referential. Another limitation concerns the costing methods used in the hospital cost accounting system and the ENC. Although bottom-up microcosting is known to be the best method to assess hospital costs, all databases analyzed in our study used a top-down microcosting approach. The latter method is certainly less accurate than bottom-up microcosting but is more easily applicable and more developed in other countries in view of future comparisons [6]. The last important limitation concerns the consideration or lack of consideration of all hospital cost accounting data in the mean cost calculation of each DRG. As shown in our previous publication, three-quarters of hospital stays for organ recovery are classified in a medical DRG according to the main cause of hospital admission. The methodology of the ENC for calculating the mean cost of each DRG excludes extreme values. Due to the surgical procedure, a hospital stay during which organ recovery occurs is often more expensive than a conventional medical DRG; thus, these stays are more likely to represent an extreme value and ultimately to be excluded from calculation of the mean cost of the DRG in which the hospital stay is classified. Moreover, as described in the Methods section, the central authority applies a methodology for discharging expenses on stays collected by hospital cost accounting and ultimately produces a full cost per stay for each DRG. These complex reprocessing operations increase the difficulty of comparing activity-based pricing with DRG pricing.

The HCL is a university hospital and is not nationally representative. Thus, generalization of this comparative study to other establishments participating in the ENC is easily achievable if the institutions comply with the common methodology recommended for their cost accounting. Such efforts would strengthen our results and ensure that they are not tied to the HCL. Generalization to other types of stays would ensure that the differences found were not related to the organ recovery procedure but rather to the use of cost data sources with different levels of aggregation. For a better comparison between hospital cost accounting and ENC data, this type of study should be conducted on a health procedure with a specific DRG code. In many developed countries, reference hospitals collect cost data that are reassembled at a higher level of aggregation within a national database [9]. The generalization of the findings of this comparative study to other countries would enable a study of the representativeness of national databases compared to local databases.

## Conclusions

The choice of cost data sources is a challenge common to all countries wishing to conduct quality medico-economic assessments. Due to the growing number of available databases, this choice is increasingly complex. Researchers are waiting for clear recommendations that will allow a choice between different data sources and thus facilitate comparisons between future national and international studies. Moreover, readers, hospital managers and decision makers require a fundamental understanding of the strengths and weaknesses of each database used to allow interpretation of the results in an informed context.

## Abbreviations

ENC: National Cost Study (Etude Nationale des Coûts)
DRGs: Diagnosis-Related Groups
HCL: Hospices Civils de Lyon
RCI: relative cost index
